# Rupture of the profunda femoris artery in a patient with alcoholic liver disease: a case report

**DOI:** 10.1186/1752-1947-7-95

**Published:** 2013-04-05

**Authors:** Philippa Orchard, Benjamin HL Tan, Kumar Abayasekara

**Affiliations:** 1 Department of Vascular Surgery, Royal Derby Hospital, Uttoxeter Road, Derby DE22 3NE, UK; 2 Division of Gastrointestinal Surgery, Nottingham University Hospitals, Queen’s Medical Centre, Nottingham NG7 2UH, UK

## Abstract

**Introduction:**

Profunda femoris artery aneurysms are rare and often present with rupture. However, to the best of our knowledge, rupture of a non-aneurismal profunda femoris artery has never been reported before.

**Case presentation:**

We report the case of a 31-year-old Caucasian man with alcoholic liver disease who presented with rupture of the profunda femoris artery following blunt trauma which was treated by endovascular embolization.

**Conclusion:**

Coagulopathy secondary to alcoholic liver disease is a major contributory factor and a high index of suspicion of vascular injury must be attached to such patients following blunt trauma. Although there have no previous documented cases, treatment by endovascular embolization appears to be effective and safe.

## Introduction

A review of the available literature reveals numerous case reports on the rupture of profunda femoral artery aneurysms. These aneurysms are rare (0.5% of all peripheral aneurysms). When they rupture, the symptoms include sudden onset of localized pain, swelling and compartment syndrome [1]. Although our patient presented with a swollen, bruised and painful leg, there was no evidence of a profunda femoris artery aneurysm in either a computed tomography (CT) angiogram or a lower limb angiogram. We present a case of a rupture of a normal profunda femoris artery, of which we believe there are no current documented cases in the literature.

## Case presentation

A 31-year-old Caucasian man with a background of alcoholic liver disease and hepatitis presented to our Emergency Department complaining of a swollen right leg and widespread bruising. He reported that it was due to play fighting with his young son. His initial blood investigations showed that he had a hemoglobin (Hb) level of 6.9g/dL, platelet count of 28 × 10^3^cells/μL and deranged clotting (prothrombin time 25s, activated partial thromboplastin time 47 seconds, international normalized ratio 2.1). He was admitted to our hospital and received a four-unit blood transfusion. His condition improved and our patient self-discharged before further investigations could be carried out.

He returned to the hospital eight days later complaining of worsening pain and swelling in his leg which was so severe he was struggling to mobilize. His leg was grossly edematous, and tender throughout with widespread bruising. His distal sensation was intact. Again he had a low Hb level (4.4g/dL) and platelet count (52 × 10^3^cells/μL) and his clotting was deranged (prothrombin time 27s, activated partial thromboplastin time 36s, international normalized ratio 2.3), so he received a further blood transfusion and intravenous vitamin K.

He was reviewed by our orthopedic team, who were unsure as to the nature of the swelling. It was noted that he had no signs of compartment syndrome, so leg elevation was recommended.

**Figure 1:**
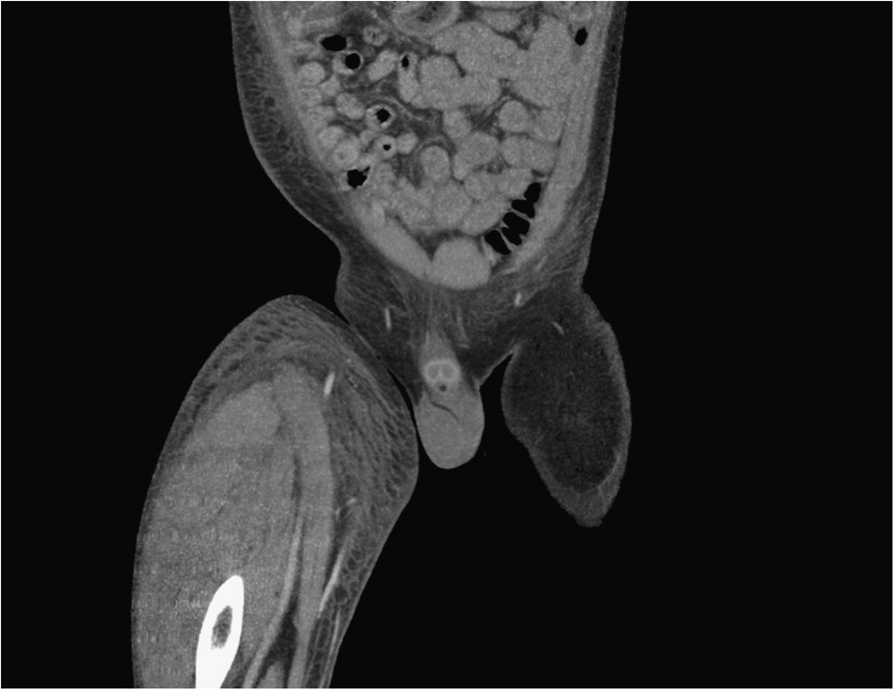
Coronal computed tomography angiogram showing a large hematoma in the lateral component of the thigh.

Over the next 24 hours, his condition failed to improve. His Hb level dropped again to 4.3g/dL and his international normalized ratio increased to 3.2. He received a further transfusion of red blood cells, fresh frozen plasma and cryoprecipitate. He was reviewed by a vascular surgeon, who arranged for a CT angiogram. This showed normal appearance of his external iliac and common femoral and superficial femoral arteries but a large hematoma in the lateral component of his thigh, with an area of high attenuation that may have represented active bleeding (Figure 1). The most likely bleeding source was a branch of his profunda femoris artery.

**Figure 2:**
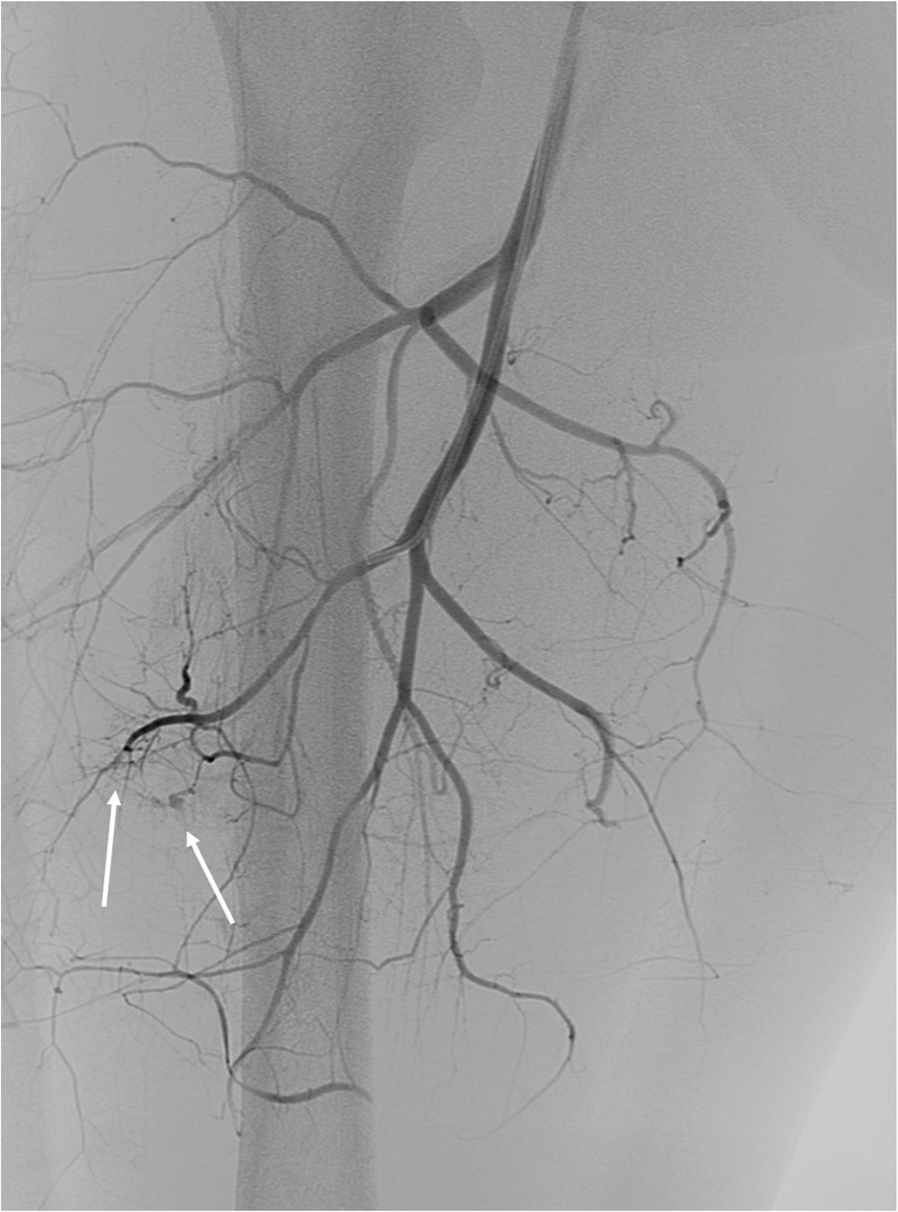
Selective right femoral angiography and superior selective images of the profunda femoris branches showing two small bleeding sources (arrows).

Following the CT scan, he had an urgent right lower limb angiogram. His left common femoral artery was punctured and a 5F sheath was positioned across the aortic bifurcation into his right common iliac artery. Selective right femoral angiography was performed and superior selective images of the profunda femoris branches obtained. Two small bleeding sources were demonstrated running laterally off the small branches of the profunda femoris (Figure 2). These were successfully embolized using a gel foam slurry (Pharmacia & Upjohn Inc., Bridgewater, New Jersey, USA).

The CT scans were reviewed with the consultant orthopedic surgeon and there was concern about compartment syndrome in his right thigh. Our patient therefore underwent a right lateral thigh fasciotomy, which was closed two days later.

Our patient made a good clinical recovery and was discharged seven days after admission. However, he failed to attend his follow-up clinic appointment.

## Discussion

Arterial bleeding following the rupture of a non-aneurysmal vessel is extremely rare. There has only been one other documented case report. The patient in that case similarly had coagulopathy secondary to alcoholic liver disease and had a spontaneous rupture of the lateral thoracic artery [2].

Coagulopathy in combination with alcoholic liver disease manifests as thrombocytopenia and thrombopathy. Approximately 40% of patients with alcoholic liver disease and cirrhosis have a prolonged bleeding time in excess of 10 minutes, and a corresponding decrease in platelet count to less than 100 × 10^3^cells/μL [3]. This has clearly been a significant contributory factor in the clinical course and presentation in our case.

As there have not been any index cases on the treatment of the rupture of a non-aneurysmal profunda femoris artery, our clinical team opted for an endovascular approach. The bleeding was successful stopped by embolization with a gel foam slurry.

## Conclusion

This is a highly rare and unusual case of the rupture of a non-aneurismal profunda femoris artery following blunt trauma. Clearly, coagulopathy secondary to alcoholic liver disease is a major contributory factor and a high index of suspicion of vascular injury must be attached to such patients following blunt trauma. Although there have no previous documented cases, treatment by endovascular embolization appears to be effective and safe.

## Consent

Written informed consent was obtained from the patient for publication of this case report and accompanying images. A copy of the written consent is available for review by the Editor-in-Chief of this journal.

## Competing interests

The authors declare that they have no competing interests.

## Authors’ contributions

PO was involved in the literature review and writing of the article. BT and KA were involved in writing and editing the article. All authors read and approved the final manuscript.
